# Regulation of Kv4.2 A-Type Potassium Channels in HEK-293 Cells by Hypoxia

**DOI:** 10.3389/fncel.2014.00329

**Published:** 2014-10-14

**Authors:** Yu-Qiang Liu, Wen-Xian Huang, Russell M. Sanchez, Jia-Wei Min, Jiang-Jian Hu, Xiao-Hua He, Bi-Wen Peng

**Affiliations:** ^1^Department of Physiology, Hubei Provincial Key Laboratory of Developmentally Originated Disorder, School of Basic Medical Sciences, Wuhan University, Wuhan, China; ^2^Department of Surgery, College of Medicine, Texas A&M Health Science Center, Neuroscience Institute, Scott and White Hospital, Central Texas Veterans Health Care System, Temple, TX, USA

**Keywords:** A-current, hypoxia, patch-clamp, *in vitro*

## Abstract

We previously observed that A-type potassium currents were decreased and membrane excitability increased in hippocampal dentate granule cells after neonatal global hypoxia associated with seizures. Here, we studied the effects of hypoxia on the function and expression of Kv4.2 and Kv4.3 α subunit channels, which encode rapidly inactivating A-type K currents, in transfected HEK-293 cells to determine if hypoxia alone could regulate I_A_ *in vitro*. Global hypoxia in neonatal rat pups resulted in early decreased hippocampal expression of Kv4.2 mRNA and protein with 6 or 12 h post-hypoxia. Whole-cell voltage-clamp recordings revealed that similar times after hypoxia (1%) *in vitro* decreased peak currents mediated by recombinant Kv4.2 but not Kv4.3 channels. Hypoxia had no significant effect on the voltage-dependencies of activation and inactivation of Kv4.2 channels, but increased the time constant of activation. The same result was observed when Kv4.2 and Kv4.3 channels were co-expressed in a 1:1 ratio. These data suggested that hypoxia directly modulates A-type potassium channels of the subfamily typically expressed in principal hippocampal neurons, and does so in a manner to decrease function. Given the role of I_A_ to slow action potential firing, these data are consistent with a direct effect of hypoxia to decrease I_A_ as a mechanism of increased neuronal excitability and promotion of seizures.

## Introduction

Voltage-dependent potassium (Kv) channels have a role in many developmental nervous system diseases, such as learning and cognitive impairment and epilepsy (Lawson, 2000; Wulff et al., 2009). A-type potassium channels are abundantly expressed in neurons and serve a number of functions, including regulation of excitability, fast-spiking, neurotransmitter release, and control of neural networks in physiological and pathophysiological processes (Birnbaum et al., 2004). A-type currents (I_A_) exhibit rapid activation, inactivation, and rapid recovery from inactivation at hyperpolarized membrane potentials (Jerng et al., 2004).

Kv channels are composed of pore-forming α proteins and auxiliary β subunits (Patel and Honore, 2001; Conforti et al., 2003a). Among the Kv4 α subunits (Kv4.1, Kv4.2, and Kv4.3), Kv4.2 and Kv4.3 underlie the somatodendritic A-type K^+^ currents in the central nervous system (CNS) (Huang et al., 2005), whereas, Kv4.1 mRNA levels are lower than Kv4.2 or Kv4.3 (Serodio and Rudy, 1998). In the hippocampus, Kv4.2 is mainly expressed in area CA1 pyramidal cells, and comprises the pore-forming subunit together with Kv4.3, whereas CA1 inhibitory interneurons express primarily Kv4.3 channels (Birnbaum et al., 2004). Down-regulation of A-type K^+^ channel function in pyramidal neuron dendrites increases neuronal excitability (Bernard et al., 2004), and selective blockade of A-type potassium channel can cause seizures (Ruschenschmidt et al., 2006).

The ability to sense and respond to hypoxia is critical for the survival of many cell types or tissues to their changing environment. In particular, O_2_-sensitive potassium channel expression has been studied in many chemosensitive cells such as carotid body, neuroepithelial body, pheochromocytoma (PC12), and pulmonary artery smooth muscle cells (Conforti et al., 2003b). Some potassium channel subtypes expressed in hypoxia-sensitive tissues have also been reported sensitive to low pO_2_ modulation in heterologous expression systems (Patel et al., 1997; Conforti et al., 2000). Recombinant Kv channels formed by several Kv α subunits or α–β interactions have been reported to exhibit sensitivity to hypoxia. These include Kv1.2, Kv1.5, Kv2.1, Kv3.1b, Kv3.3, Kv1.2/Kv1.5, Kv2.1/Kv9.3, and Kv4.2/kvβ1.2 (Wang et al., 1996; Patel et al., 1997; Archer et al., 1998, 2001; Hulme et al., 1999; Perez-Garcia et al., 1999, 2000; Conforti et al., 2000; Osipenko et al., 2000; Lopez-Barneo et al., 2001). Co-expression of Kvβ1.2 has been reported to confer oxygen sensitivity to Kv4.2 but not Kv1.3 channels in HEK-293 cells (Perez-Garcia et al., 1999). Furthermore, expression of a dominant-negative Kv4 construct (Kv4.xDN) can suppress the hypoxia-induced depolarization in chemoreceptive cells, suggesting direct effects of hypoxia on the Kv4 channel subfamily (Perez-Garcia et al., 2000). Notably, hypoxia can inhibit O_2_-sensitive K^+^ channels via both inhibition of channel activity and down-regulation of the channel expression (Yuan, 2001; Conforti et al., 2003a).

We previously reported that A-type K^+^ currents were significantly decreased and membrane excitability was increased in hippocampal dentate granule cells after seizure-inducing hypoxia *in vivo* (Peng et al., 2013). However, the mechanisms that mediated this hypoxic inhibition remain to be elucidated. In the current study, we therefore investigated the mRNA and protein expression levels of the Kv4.2 and Kv4.3 after hypoxia treatment in rats. We further expressed Kv4.2 and co-expressed Kv4.2/Kv4.3 in HEK-293 cells to investigate a potential direct influence of hypoxia on the function of A-type channels.

## Materials and Methods

### *In vivo* hypoxia treatment

Animals were obtained from the Animal Biosafety Level 3 Laboratory (ABSL-3) of Wuhan University. All experimental protocols were approved by the Committee on the Ethics of Animal Experiments of the Wuhan University (China). Rat pups on postnatal day 10 were removed from the litter and placed on a heating pad in a custom-made acrylic glass chamber with an O_2_ sensor mounted inside and three gas inlets for N_2_ infusion (Peng et al., 2013). For the treated pup, the chamber O_2_ concentration was lowered by N_2_ infusion to 7% within 30–40 s, maintained at 6–7% for an additional 4 min, lowered to 5–6% for 8 min, and then lowered by 1% per minute until the pup became apneic for 30 s, at which time the chamber lid was removed for exposure to room air. The total time of hypoxia exposure was 14–16 min. Using this approach, hypoxia-treated pups typically exhibited spontaneous convulsive seizures lasting 10–60 s beginning 2–4 min after hypoxia onset, which recurred throughout hypoxia exposure and continued for several minutes after return to room air. Littermate controls were kept at room air and maintained body temperature by heating pad. All rats were immediately returned to their dam after the experiment. Animals were sacrificed for the brain sample at 6, 12, and 24 h after hypoxia treatment.

### Quantitative real time PCR analysis

Total RNA was isolated from the whole hippocampus of rat using TRIzol Reagent (Invitrogen, Carlsbad, CA, USA) followed by chloroform extraction and isopropanol precipitation. RNA was quantified by spectrophotometric absorbency at 260 nm, purity confirmed by A260/A280 ratio. RNA samples were stored at −80°C.

Kv4.2 and Kv4.3 cDNA was synthesized using 2 μg of total RNA and a reverse transcription kit (Fermentas Canada Inc., Burlington, ON, Canada) with a 20 μl reaction system. Quantitative PCR (BioRadMyIQ5; Life Technologies, Grand Island, NY, USA) was performed in a 0.2 ml PCR tube (Axygen; VWR International, Radnor, PA, USA) containing 1 μL of cDNA, 0.5 μL of 10 μmol/L of each primer, 10 μL of a 2 × PCR mixture (All-in-One qPCR Mix; GeneCopoeia, Inc., Rockville, USA), 8 μL of diethylpyrocarbonate H_2_O for a total volume of 20 μL. The reaction conditions for the quantitative PCR were 95°C for 10 min, 95°C denaturation for 10 s, 58°C renaturation for 20 s, and 72°C extension for 25 s for a total of 40 cycles. Fluorescence was collected at 72°C. The temperatures of the melting curve ranged from 72 to 95°C, collecting fluorescence every 0.5°C for 47 cycles. The PCR primers for Kv4.2 were as follows: 5′-GTGTCAGGAAGTCATAGAGGC-3′ (forward) and 5′-TTACAAAGCAGACACCCTGA-3′ (reverse). The primers for Kv4.3 were 5′-CACCACCTGCTACACTGCTTAGAA-3′ (forward) and 5′-TCTGCTCATCAATAAACTCGTGGTT-3′ (reverse). The reference gene used was β-actin, and the two primers for this sequence were as follows: (forward) 5′-CACGATGGAGGGGCCGGACTCATC-3′; (reverse) 5′-TAAAGACCTCTATGCCAACACAGT-3′. The data were analyzed using 2^−ΔΔCt^ method to compare all groups in our experiments.

### Western blot

The samples were extracted from the whole hippocampus, homogenized with 2% SDS, 100 mM dithiothreitol, and 10% glycerol and stored at 4°C. Equal amount of proteins were loaded and separated on 10% SDS-PAGE. Then the proteins were transferred to a polyvinylidene fluoride (PVDF) membrane and blocked by immersion for 2 h in Tris-buffered saline (TBS) containing 5% dry milk. The membranes were then incubated at 4°C overnight with rabbit polyclonal anti-Kv4.2 (Sigma, USA; used at 1:1000) and Kv4.3 receptor antibodies (Sigma, USA; used at 1:1000) and β-actin rat monoclonal antibody (Tianjin Sungene Biotech Co., Ltd, Tianjin, China; used at 1:2000). After incubation, the membranes were washed three-times with TBST (TBS containing 0.2% Tween-20) and were then incubated again for 2 h with the goat anti-rat IgG (diluted 1:20,000) at room temperature. The membranes were washed again with TBST three-times. Finally, the reaction was developed using a chemiluminescent reagent (ECL; Ecl Advantage Inc., Menlo Park, CA, USA) and exposed to Hyper film (GE Healthcare Life Sciences, Pittsburgh, PA, USA).

### Cell transfections and treatment with hypoxia

Cell culture was carried out using standard procedures. HEK-293 cells were maintained in DMEM supplemented with 10% fetal calf serum (GIBCO BRL) and incubated in a humidified atmosphere containing 5% CO_2_ in air at 37°C. For transfections, 1 μg of pcDNA3.0-Kv4.2 or pcDNA3.0-Kv4.2/Kv4.3 DNA into HEK-293 cells was performed using Lipofectamine 2000 (Invitrogen) according to the instruction of the manufacturer. All experiments were recording HEK-293 cells within 20 passages. To study the effect of hypoxia on K^+^ currents, cells after transfected were maintained in a modular incubator chamber (Billups-Rothenberg, Del Mar, CA, USA) at 37°C with 1% O_2_ for 6 or 12 h. Cells transfected for 24 h total were used for electrophysiological recordings, with hypoxia exposure carried during the final 6 or 12 h of incubation.

### Electrophysiological recordings

K^+^ currents were studied using the whole-cell patch-clamp technique and voltage-clamp recordings were carried out at room temperature (22–25°C) by using a MultiClamp-700B amplifier and Digidata-1440A A/D converter (Axon Instruments, CA, USA). Recording electrodes were pulled using a Sutter P-97 puller (Sutter Instruments, CA, USA). Electrodes were filled with intracellular solution containing (in millimolar): 125 KCl, 4 MgCl_2_, 10 HEPES, 10 EGTA, and 5 MgATP, adjusted to 310 mOsm/L with sucrose, pH 7.2 with KOH. The external solution contained (in millimolar): 141 NaCl, 4.7 KCl, 1.2 MgCl_2_, 1.8 CaCl_2_, 10 glucose, and 10 HEPES, adjusted to 330 mOsm/L with sucrose, pH 7.4 with NaOH. The resistance of the recording pipette filled with internal solution was 2–6 MΩ. The adjustment of capacitance compensation and series resistance compensation was done before recording the membrane currents. The holding potential was −80 mV. Isolated HEK-293 cells were studied 24 h after transfection. Membrane currents were filtered at 2 kHz and digitized at 10 kHz, and the data were stored in compatible PC computer for off-online analysis using the pCLAMP 10 acquisition software (Axon Instruments, CA, USA).

The voltage-dependence of activation was studied by a 400 ms constant depolarizing pulse from −50 mV to +40 mV in 10 mV steps (see Figure 2A inset). To determine the voltage-dependence of steady-state inactivation of Kv4.2, the voltage-dependence of inactivation was assessed by measuring the peak amplitude of current responses evoked by test depolarization to +40 mV after 1.5 s pre-pulse to potentials between −110 and 0 mV with 10 mV increment (see Figure 2B inset).

### Data analysis

All data were analyzed by Igor Pro 6.10 (Wavemetrics, Lake Oswego, OR, USA), pCLAMP 10 (Axon Instruments, CA, USA) and Origin 7.5 (Microcal Software, USA). To construct steady-state activation curves, peak currents (*I*) were converted into conductance (*G*) using the formula: *G* = *I*/(V_m_−V_rev_), where V_m_ is the membrane potential, and V_rev_ is the reversal potential (−90 mV in our experiments). The normalized conductance was fitted by a Boltzmann Equation: *G*/Gmax = 1/{1 + exp [(V_m_−V_1/2_)/*k*]}, where V_1/2_ is the half-maximal membrane potential for activation and *k* is the slope factor. The steady-state inactivation curves were established using a Boltzmann equation: *I*/Imax = 1/{1 + exp[(V_m_−V_1/2_)/*k*]}, where V_1/2_ is the half-maximal membrane potential for inactivation and *k* is the slope factor. Mono- or bi-exponential functions were used to fit current rise time and decay time, respectively, using one of the following equations: *y*(*t*) = *A*_1_exp(−*t*/τ) + *B* or *y*(*t*) = *A*_1_exp(−*t*/τ_1_) + *A*_2_exp(−*t*/τ_2_) + *B*, where *t* is time, *A*_1_ and *A*_2_ are the amplitudes, and τ_1_ and τ_2_ are the time constants of decay.

All data reported in this study are expressed as means ± SEM, Statistical significance was verified using one-way ANOVA test and Student’s *t*-test. *P* < 0.05 was the evaluation criterion for statistical significance.

## Results

### Hypoxia treatment decreased Kv4.2 expression in rat hippocampus

**Figure 1:**
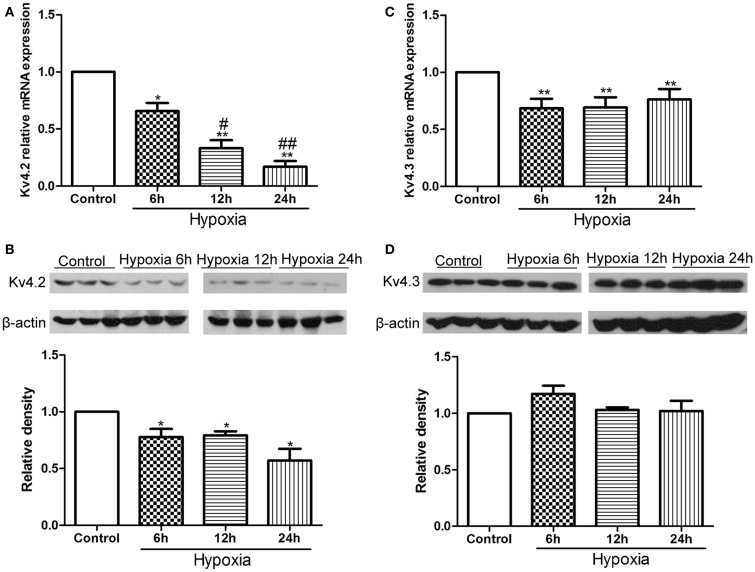
**Effect of hypoxia on the mRNA and protein levels of Kv4.2 and Kv4.3**. Quantification of mRNA levels of Kv4.2 **(A)** and Kv4.3 **(C)**. Representative image and quantification of protein levels of Kv4.2 **(B)** and Kv4.3 **(D)** in control and hypoxia groups. Equal amounts of collected protein samples were subjected to Western immunoblot analysis. β-Actin was used as an internal control. Data are presented as means ± SEM (*n* = 3 for protein and mRNA in each group). *p < 0.05, **p < 0.01 vs. control. ^#^*p* < 0.05, ^##^*p* < 0.01 vs. hypoxia 6 h.

First, we examined the expression of Kv4.2 and Kv4.3 after hypoxia treatment in neonatal rat pups. Kv4.2 and Kv4.3 mRNA and protein from hippocampal tissue before or at 6, 12, and 24 h after hypoxia treatment were analyzed by qPCR and western blot (Figure 1). The levels of Kv4.2 mRNA and protein were significantly decreased by hypoxia treatment at each time point we tested (Figures 1A,B). 6, 12, and 24 h after the hypoxia treatment, Kv4.2 mRNA was decreased by 35% (*n* = 3, *p* < 0.05 vs. control), 68% (*n* = 3, *p* < 0.01 vs. control), and 84% (*n* = 3, *p* < 0.01 vs. control), respectively, and Kv4.2 protein was decreased by 22% (*n* = 3, *p* < 0.05 vs. control), 21% (*n* = 3, *p* < 0.05 vs. control), and 43% (*n* = 3, *p* < 0.01 vs. control), respectively. The hypoxia-induced reduction of Kv4.2 mRNA was clearly time-dependent (Figure 1A), with a significant reduction observed 6 h after hypoxia treatment, and a larger reduction at 12 and 24 h post-hypoxia exposure. However, there was no marked difference between 12 and 24 h post-hypoxia treatment. In contrast, Kv4.3 mRNA was comparably decreased across all time points tested, with no further decrease up to 1 day post-hypoxia (Figure 1C). Notably, Kv4.3 protein was not significantly altered within 24 h after hypoxia treatment (Figure 1D).

### Hypoxicsuppression of Kv4.2 channel-mediated current

**Figure 2:**
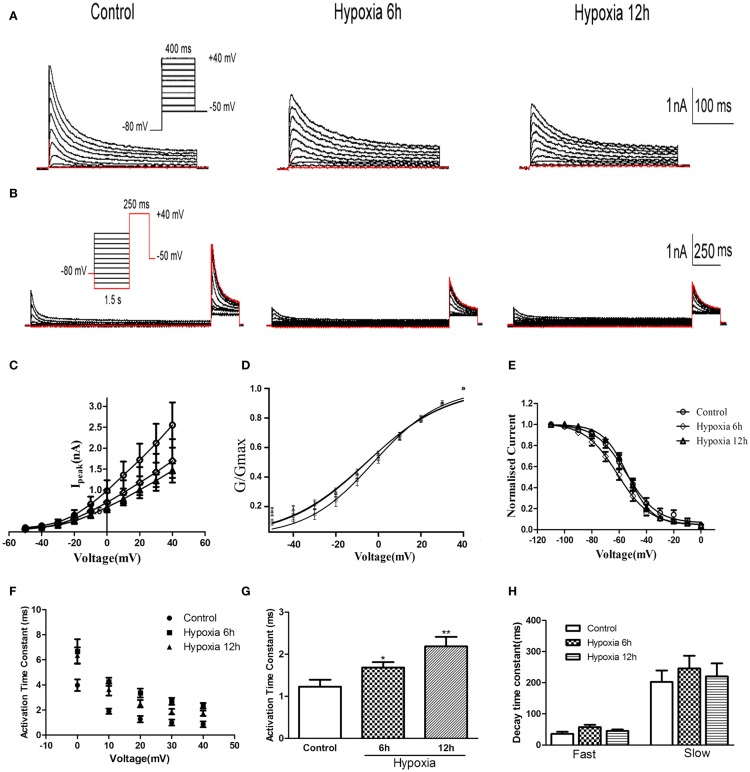
**Voltage-dependence of activation and inactivation of transient currents at 6, 12 h after hypoxia in the HEK293 cells**. **(A)** Superimposed current traces evoked by depolarizing steps to potentials between −50 and +40 mV with 10 mV increment after −80 mV. **(B)** Superimposed current traces evoked by test depolarization to +40 mV after 1.5 s pre-pulse to potentials between −110 and 0 mV with 10 mV increment. **(C)** Averaged I–V relationship obtained in the HEK293 cells. The peak currents were reduced by hypoxia treatment. Each curve represents the Mean ± SEM of 10–15 cells. **(D)** Mean normalized conductance–voltage relations for the peak outward Kv4.2 currents recorded in HEK293. **(E)** Voltage-dependences of steady-state inactivation of Kv4.2-induced K^+^ currents in HEK293. **(F)** Activation time constants of Kv4.2 currents were determined from single exponential fits to the rising phases of the currents. The activation time constants are voltage-dependent. **(G)** The activation time constants (τ) (evoked at +40 mV) were increased after hypoxia treatment. Mean ± SEM was displayed. **(H)** Decay time constants of Kv4.2 (evoked at +40 mV) in HEK293 cells were fitted by bi-exponential function. Both of the fast and slow-decay time constant of kv4.2 currents were not significantly change after hypoxia treatment. Mean ± SEM was displayed. *p < 0.05, **p < 0.01.

Whole-cell patch-clamp recording was performed on HEK-293 cells transfected with Kv4.2. As shown in Figures 2A–C, Kv4.2 channel currents exhibited rapid activation and inactivation typical of A-type potassium currents. Since there was no significant difference in mRNA and protein between 12 and 24 h after hypoxia treatment *in vivo*, we examined the properties of Kv4.2 channel currents at only 6 and 12 h after hypoxia treatment. Peak Kv4.2 channel-mediated current amplitudes were significantly decreased at 6 and 12 h after hypoxia treatment. The peak current amplitude was 2.55 ± 0.54 nA (*n* = 10) at +40 mV in the control group and 1.70 ± 0.52 nA (*n* = 10, *P* < 0.01) and 1.46 ± 0.17 nA (*n* = 13, *P* < 0.01) at 6 and 12 h after hypoxia treatment, respectively (Figure 2C).

We next investigated the effect of hypoxia on Kv4.2 channel gating properties (Figure 2). Figure 2D shows that hypoxia did not significantly alter the voltage-dependent activation of Kv4.2 channels. Single Boltzmann distribution fits showed that Kv4.2 channels under control conditions exhibited a voltage of half-maximum activation (V_1/2_) of V_1/2_ = −3.26 ± 0.96 mV and a *k*_act_ of 15.95 ± 0.89 mV (*n* = 7). No significant changes in V_1/2_ and *k* were detected at 6 and 12 h after hypoxia (V_1/2_ = −5.42 ± 1.56 mV, *k* = 18.71 ± 1.51 mV, *n* = 8; V_1/2_ = −5.59 ± 1.64 mV, *k* = 19.12 ± 1.60 mV, *n* = 11, respectively) (Table 1). For steady-state inactivation (Figure 2E), the V_1/2_ of the inactivation removal also did not shift significantly, and was −54.66 ± 1.48 mV (*n* = 6) for controls vs. −55.96 ± 1.09 mV (*n* = 8, *P* > 0.05, 6 h hypoxia vs. control) and −53.84 ± 0.72 mV (*n* = 7, *P* > 0.05 12 h hypoxia vs. control) after 6 and 12 h hypoxia, respectively (Table 1). The activation slope factor (*k*) also was not significantly different between control and hypoxia groups.

**Table 1:** **Comparison of properties of Kv4-induced K^+^ currents**.

	Control		Hypoxia 6 h		Hypoxia 12 h	
	Kv4.2	Kv4.2/Kv4.3	Kv4.2	Kv4.2/Kv4.3	Kv4.2	Kv4.2/Kv4.3
V_1/2_ activation (mV)	-3.26 ± 0.96	-10.25 ± 0.65	-5.42 ± 1.56	-7.36 ± 0.70	-5.59 ± 1.64	-7.48 ± 0.80
*k*_act_	15.95 ± 0.89	15.97 ± 0.60	18.71 ± 1.51	15.89 ± 0.64	19.12 ± 1.60	15.961 ± 0.74
Activation T (ms) (+40 mV)	1.23 ± 0.17	0.97 ± 0.04	1.68 ± 0.13*	2.53 ± 0.23***	2.19 ± 0.22**	1.99 ± 0.23***
V_1/2_ inactivation (mV)	-54.66 ± 1.48	-42.07 ± 0.72	-55.96 ± 1.09	-45.58 ± 0.74	-53.84 ± 0.72	-49.37 ± 0.73
*k*_inact_	-0.04 ± 0.005	-0.07 ± 0.007	-0.04 ± 0.003	-0.06 ± 0.005	-0.05 ± 0.003	-0.05 ± 0.004
Inactivation T (ms)	35.97 ± 6.36	36.48 ± 1.99	57.45 ± 7.39	49.63 ± 5.35	44.64 ± 4.97	35.97 ± 6.36
(+40 mV)	202.60 ± 36.78	200.80 ± 22.28	245.90 ± 40.84	233.50 ± 46.28	220.70 ± 41.49	213.00 ± 35.91
Recovery τ (ms)	94.84 ± 9.94	37.26 ± 4.83	91.24 ± 12.9	93.15 ± 15.22***	86.32 ± 8.36	66.55 ± 7.44**

All data were obtained at room temperature (20–22°C) and are expressed as Mean ± SEM of 10 to 15 cells from each group. *p < 0.05, **p < 0.01, ***p < 0.001, all compared with control.

To characterize the rate of activation of the Kv4.2-induced currents in HEK-293 cells, the rising phases of Kv4.2 currents evoked at test potentials between 0 and +40 mV were fitted by single exponential function after the onset of the voltage step to the peak of the outward current. As is evident, the Kv4.2-induced currents activate rapidly, and the activation time constants vary with voltage, decreasing with increasing membrane depolarization (Figure 2F). However, the Kv4.2-induced currents activate more slowly after hypoxia treatment. Figure 2G showed that the activation time constant evoked at +40 mV in control was increased at 6 and 12 h after hypoxia treatment from 1.23 ± 0.17 ms (*n* = 6) to 1.68 ± 0.13 ms (*n* = 7, *p* < 0.05 vs. control) and 2.19 ± 0.22 ms (*n* = 10, *p* < 0.01 vs. control), respectively (Table 1). Next, we investigated the decay kinetics of Kv4.2 currents evoked at +40 mV. The decay phase of the currents could be well fitted by a bi-exponential function, yielding fast and slow-decay time constants. As shown in Figure 2H, neither τ_fast_ nor τ_slow_ displayed a significant change after hypoxia treatment.

**Figure 3:**
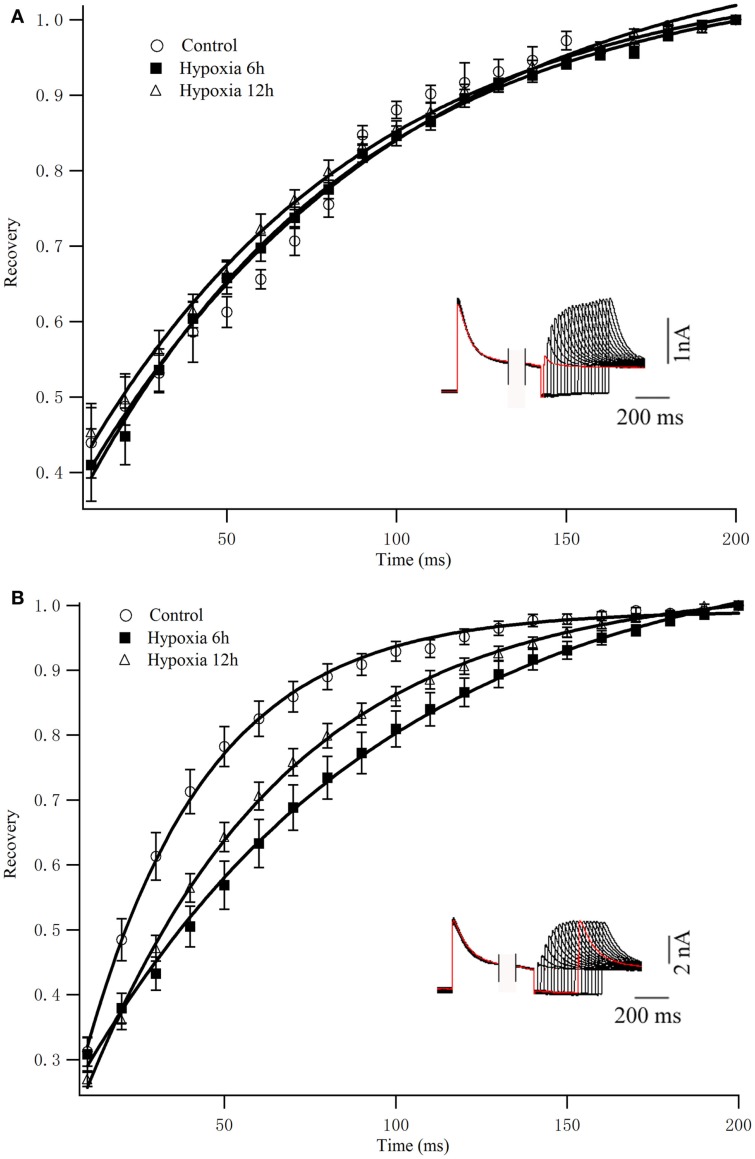
**Recovery from inactivation of Kv4.2 and co-expression of Kv4.2/Kv4.3**. **(A)** Inactivation recovery was examined by inactivating Kv4.2 **(A)** and Kv4.2/Kv4.3 **(B)** current and then stepping to −110 mV for increasing before a test step to 40 mV. Mean ± SEM normalized currents in HEK-293 (*n* = 10) cells are plotted as a function of recovery time. Data points were fitted with mono-exponential. Inset represents the exemplificative experimental traces of Kv4.2 **(A)** and Kv4.2/Kv4.3 **(B)** in control group.

To determine the rates of Kv4.2 recovery from steady-state inactivation, cells were first depolarized to +40 mV for 1 s (to inactivate the currents), then hyperpolarized to −110 mV for varying times (ranging from 10 to 200 ms) prior to test depolarizations to +40 mV (to assess recovery). Recovery curves were generated by plotting normalized current amplitudes (normalized to maximum) of activation as a function of pre-pulse time and fitting with a single exponential. At −110 mV, the recovery time constant in control was 94.84 ± 9.94 ms (*n* = 8) (Figure 3A). The recovery time constant at 6, 12 h after hypoxia treatment were not significantly changed (91.24 ± 12.90 ms, *n* = 10, *p* > 0.05, 6 h vs. control; 86.32 ± 8.36 ms, *n* = 10, *p* > 0.05, 12 h vs. control) (Table 1). Therefore, except for the time to activation, most biophysical features of Kv4.2 currents remained unchanged after hypoxia.

### Effects of hypoxia on heteromeric Kv4.2/Kv4.3 channels in HEK-293 cells

**Figure 4:**
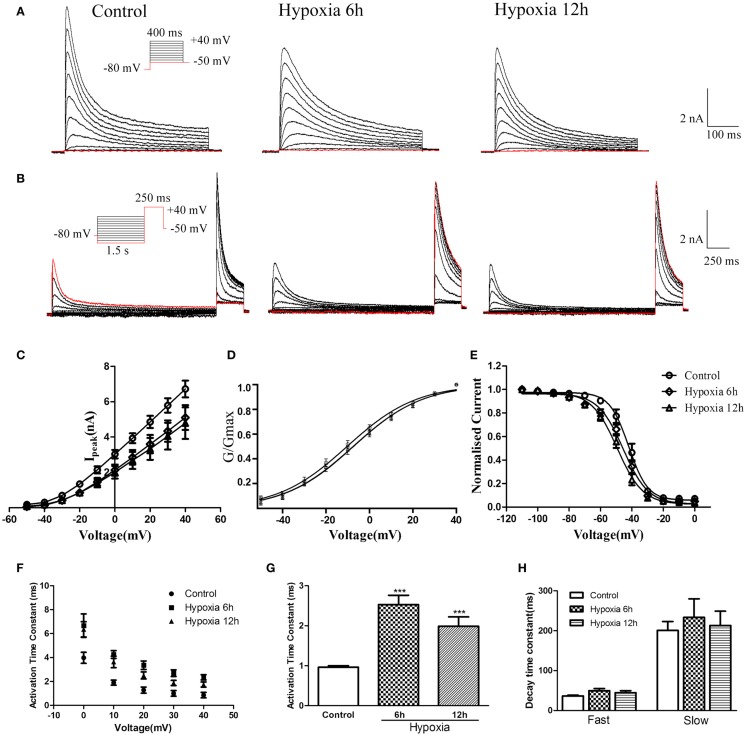
**Voltage-dependence of activation and inactivation of transient currents Kv4.2/Kv4.3 at 6, 12 h after hypoxia in the HEK293 cells**. **(A)** Superimposed current traces evoked by depolarizing steps to potentials between −50 and +40 mV with 10 mV increment after −80 mV. **(B)** Superimposed current traces evoked by test depolarization to +40 mV after 1.5 s pre-pulse to potentials between −110 and 0 mV with 10 mV increment. **(C)** Averaged I–V relationship obtained in the HEK293 cells. The peak currents were reduced by hypoxia treatment. Each curve represents the mean ± SEM of 10–15 cells. **(D)** Mean normalized conductance–voltage relations for the peak outward Kv4.2/Kv4.3 currents recorded in HEK293. **(E)** Voltage-dependences of steady-state inactivation of Kv4.2/Kv4.3-induced K^+^ currents in HEK293. **(F)** Activation time constants of Kv4.2/Kv4.3 currents were determined from single exponential fits to the rising phases of the currents. The activation time constants are voltage-dependent. **(G)** The activation time constants (τ) (evoked at +40 mV) were increased after hypoxia treatment. Mean ± SEM was displayed. **(H)** Decay time constants of Kv4.2 (evoked at +40 mV) in HEK293 cells were fitted by bi-exponential function. Both of the fast and slow-decay time constant of Kv4.2/Kv4.3 currents were not significantly change after hypoxia treatment. Mean ± SEM was displayed. *p < 0.05, **p < 0.01.

Although the experiments described above revealed that hypoxia altered mRNA and protein levels of Kv4.2 and the Kv4.3 mRNA, it remained unclear whether hypoxia could affect the function of heteromeric Kv4.2/Kv4.3 channels. To determine the biophysical properties of heteromeric Kv4.2/Kv4.3 channels, Kv4.2 + Kv4.3 (in a ratio of 1:1) and EGFP were induced into HEK-293 cells, and whole-cell recordings were obtained from EGFP-positive cells 24 h after transfection. As shown in Figures 4A,B, co-expression of Kv4.2 and Kv4.3 resulted in rapidly activating and inactivating K^+^ currents typical of I_A_. The amplitudes and voltage-dependence of activation and inactivation were examined as described above for Kv4.2. Similarly, peak currents (at +40 mV) were significantly decreased at 6 and 12 h after hypoxia treatment compared to controls [Control = 6.72 ± 0.47 nA (*n* = 8); 6 h Hypoxia = 5.11 ± 0.70 nA, *n* = 8, *p* < 0.01 vs. control; and 12 h Hypoxia = 4.79 ± 0.89 nA, *n* = 9, *p* < 0.01 vs. control; Figure 4C].

Similar to the effect on Kv4.2 activation, hypoxia did not significantly alter the voltage-dependence of activation for Kv4.2/Kv4.3 channels (Figure 4D). The V_1/2_ of Kv4.2/Kv4.3 currents in the control group was −10.25 ± 0.65 mV (*n* = 8) compared to −7.36 ± 0.70 mV (*n* = 7, *p* > 0.05 vs. control) and −7.48 ± 0.80 mV (*n* = 8, *p* > 0.05 vs. control) at 6 and 12 h after hypoxia treatment, respectively (Table 1). The activation slope factor (*k*) was also not significantly different between control and hypoxia groups. Hypoxia had no significant effect on the inactivation of Kv4.2/Kv4.3 channels (Figure 4E). The inactivation V_1/2_ and *k* of Kv4.2/Kv4.3 was not significantly different between control and hypoxia groups (Control: V_1/2_ = −42.07 ± 0.72 mV, *k* = −0.07 ± 0.007 mV, *n* = 8; 6 h: V_1/2_ = −45.58 ± 0.74 mV, *k* = −0.06 ± 0.005 mV, *n* = 8, *p* > 0.05 vs. control; 12 h: V_1/2_ = −49.37 ± 0.73 mV, *k* = −0.05 ± 0.004 mV, *n* = 8, *p* > 0.05 vs. control) (Table 1).

In addition, the rising and decay phases of the Kv4.2/Kv4.3 currents were described by single or double exponentials. Similar to the results for Kv4.2 (Figure 2F), the Kv4.2/Kv4.3-induced currents activate rapidly, and the activation time constants vary with voltage, decreasing with increasing membrane depolarization (Figure 4F). However, the Kv4.2-induced currents activate more slowly after hypoxia treatment. At +40 mV, the activation time constant in the control group was significantly increased at 6 and 12 h after hypoxia treatment from 0.97 ± 0.04 ms (*n* = 10) to 2.53 ± 0.23 ms (*n* = 8, *p* < 0.001 vs. control) and 1.99 ± 0.23 ms (*n* = 7, *p* < 0.001 vs. control), respectively (Figure 4G) (Table 1). However, hypoxia had no significant effect on the fast and slow-decay time constants (Figure 4H).

To determine the rates of heteromeric Kv4.2/Kv4.3 recovery from steady-state inactivation, we used a same depolarized step protocol as in Figure 3A. The recovery curves were fitted with mono-exponential function. Notably, co-expression of Kv4.2 and Kv4.3-induced currents recovered faster from steady-state inactivation than Kv4.2-mediated currents (Figure 3B). However, the inactivation recovery rates for heteromeric Kv4.2/Kv4.3-mediated currents were significantly slowed after hypoxia treatment compared to control (*p* < 0.01) (Table 1).

## Discussion

The current study showed that hypoxia down-regulated I_A_ *in vitro*. In addition, hypoxia increased the time constant of activation without significantly altering the voltage-dependence of activation and inactivation. Furthermore, hypoxia had similar effects on co-expressed Kv4.2 and Kv4.3 channels. Importantly, we also observed that hypoxia *in vivo* decreased the expression of Kv4.2 and Kv4.3 in the hippocampus, providing a possible mechanism of I_A_ inhibition observed previously after global hypoxia [see Patel et al. (1997)].

Neuronal ion channels can have complex roles in hypoxia-induced injury. Some ion channels are up-regulated by hypoxia such as Kv2.1 (Misonou et al., 2005; Ito et al., 2010), but some are down-regulated such as Ca-dependent K^+^ (K_Ca_) channels, ATP-sensitive potassium (K_ATP_) channel, voltage-sensitive sodium channels (VSSCs), voltage-gated Ca^2+^ channels (VGCCs) (Melamed-Frank et al., 2001; Gao and Fung, 2002; Banasiak et al., 2004; Chao and Xia, 2010).

As expected, heterologously expressed Kv4.2 and Kv4.3 α subunits showed properties of I_A_ (rapid activation and inactivation) (Birnbaum et al., 2004). Previous studies have shown that I_A_ can be regulated by hypoxia/ischemia, but the functional significance of this change is speculative (Cummins et al., 1991; Hyllienmark and Brismar, 1996; Gebhardt and Heinemann, 1999; Chi and Xu, 2000). I_A_ and other K^+^ currents were transiently increased after ischemia/reperfusion *in vivo* (Chi and Xu, 2000), yet brain slices exposed to hypoxia *in vitro* resulted in inhibition of K^+^ currents (Cummins et al., 1991; Hyllienmark and Brismar, 1996; Gebhardt and Heinemann, 1999). Consistent with the latter, our data showed that I_A_ was decreased after hypoxia treatment in an *in vitro* expression system. One possible mechanism underlying the reduction of potassium currents is that the number of K^+^ channels decreased after hypoxia treatment. Kv1.3 protein levels were observed to decrease in Jurkat T cells after hypoxia, with the hypoxic down-regulation of Kv1.3 expression depending on hypoxia severity, indicating that the number of Kv channels could be altered by hypoxia (Conforti et al., 2003a). Moreover, hypoxia down-regulated the transcription and expression of Kv1.2 and Kv1.5 in rat pulmonary arterial smooth muscle cells (PASMC) (Wang et al., 1997). The human embryonic kidney cells (HEK-293) have been widely used as one mammalian expression system in the study of Kv channels. It’s important to acknowledge the possibility that endogenous K channels could contribute to currents measured through the heterologously expressed Kv channels in these cells. However, HEK-293 do not endogenously express I_A_-encoding genes (Kv1.4, Kv3.3, Kv3.4, and Kv4.1) (Jiang et al., 2002; Thomas and Smart, 2005). Also, as a blank control, we recorded from HEK-293 cells that were transfected with the reporter gene GFP without the Kv subunits (same protocol as shown in Figure 2A). The average amplitude of the depolarization-activated current was <500 pA, with a slow-activation phase and slow-decay phase, very different from A-type potassium channel (as shown in Figure S1 in Supplementary Material). Thus, in the current study, we demonstrated that Kv4.2 and Kv4.3 mRNA and protein were decreased by hypoxia, supporting decreased channels as a mechanism of I_A_ inhibition.

Another possibility is that channel properties, such as the open probability and opening time, were altered after hypoxia treatment. The redox state of amino acid residues in channel proteins is a potent regulator of channel activity for multiple ion channel types (Bahring et al., 2001; Su et al., 2007). Furthermore, Kvβ1.2 could regulate the redox and oxygen sensitivity of the Kv4.2 currents (Perez-Garcia et al., 1999). Thus, redox modulation resulting from hypoxia could be a mechanism of altered channel function.

However, the decrease of I_A_ after hypoxia treatment in the current study is different from previous studies on I_A_ during hypoxia. Deng et al. (2011) reported that I_A_ was up-regulated in ischemia-resistant large aspiny neurons and other studies have shown increased potassium currents during hypoxia (Leblond and Krnjevic, 1989). Variation in the results of previous reports and our observations could be attributed to multiple experimental variables that include the preparation (brain slice vs. cultured cells), the time and severity of hypoxia, and the comparison of native versus recombinant channels. Nonetheless, our data provide evidence that Kv4 channel function can be directly modulated by hypoxia in a manner to decrease I_A_ and promote membrane excitability.

A-type potassium currents play a critical role in action potential repolarization and inhibition of burst firing in many types of neurons and cardiac muscle (Castro et al., 2001; Kim et al., 2005). I_A_ in neuronal dendrites also contributes to the resting membrane potential and regulates firing frequency (Birnbaum et al., 2004). The reduction of I_A_ by hypoxia could increase action potential half-widths and shorten the latency to the first action potential compared to physiological conditions (Peng et al., 2013). This shortened latency also increases the probability of action potential generation in response to synaptic excitation and can change a repetitive firing pattern to burst firing, thus increasing the excitability of the neuron. Further studies have indicated that the recovery from inactivation of I_A_ can be altered by hypoxia (Ruschenschmidt et al., 2006). Although this could functionally inhibit I_A_, our previous study indicated that the speed of recovery from I_A_ inactivation was not altered by hypoxia (Peng et al., 2013).

The signaling mechanisms for I_A_ inhibition by hypoxia in the current study are speculative. Activation of PKA or PKC can shift the activation curve of I_A_ in a depolarizing direction in hippocampal area CA1 dendrites (Birnbaum et al., 2004). Furthermore, the inhibition of I_A_ induced by glial cell line-derived neurotrophic factor (GDNF) is mediated by ERK/MAPK (Yang et al., 2001). Kv4.2 is expressed on dendrites in CA1 pyramidal neurons and likely forms the pore-forming subunits of I_A_ channels in this area (Jerng et al., 2004). It is worth noting that Kv4.2 is a substrate for ERK *in vitro* and in hippocampal pyramidal neurons (Adams et al., 2000), so Kv4.2 is a target for regulation by ERK/MAPK. It is likely that kinase phosphorylation of Kv4.2 decreases the probability of channel opening or the number of channels in the dendritic membrane, and thus, this could have been a mechanism of decreased I_A_.

In summary, the current study showed a down-regulation of Kv4.2 mRNA and protein and Kv4.3 channels mRNA after hypoxia treatment *in vitro*, consistent with decreased channel expression after seizure-inducing hypoxia in neonatal rat *in vivo*. Brief hypoxia exposure induced the inhibition of peak I_A_. In addition, the time constant of activation of I_A_ was increased by hypoxia. The down-regulation of A-type channels by hypoxia may play a role in the development of neonatal seizures that result from an hypoxic episode, and our data raised possible mechanisms of direct Kv4 channel regulation by hypoxia.

## Conflict of Interest Statement

The authors declare that the research was conducted in the absence of any commercial or financial relationships that could be construed as a potential conflict of interest.

## Supplementary Material

The Supplementary Material for this article can be found online at http://www.frontiersin.org/Journal/10.3389/fncel.2014.00329/abstract
