# Supplementary material for: Regulation of Kv4.2 A-Type Potassium Channels in HEK-293 Cells by Hypoxia
Source: Front Cell Neurosci. 2014 Oct 14;8:329. doi: 10.3389/fncel.2014.00329 (PMC4196569; doi:10.3389/fncel.2014.00329)
Supplement: Supplementary file 1 [file Image_1.PDF]

Supplementary Figure

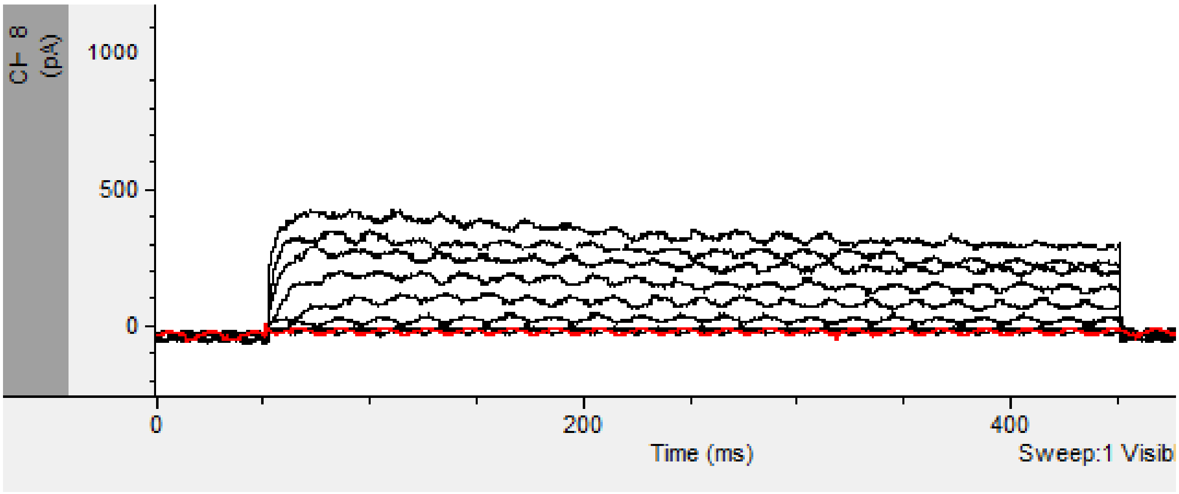

Figure S1

GFP-transfected current. It was recorded from HEK 293 cells that were transfected with the reporter gene GFP without the Kv subunits.
